# Biochemical and toxicological effect of diazepam in stress-induced cardiac dysfunctions

**DOI:** 10.1016/j.toxrep.2020.06.004

**Published:** 2020-06-18

**Authors:** Fahad A. Al-Abbasi, Vikas Kumar, Firoz Anwar

**Affiliations:** a Department of Biochemistry, Faculty of Science, King Abdulaziz University, Saudi Arabia; b Department of Pharmaceutical Sciences, Shalom Institute of Health and Allied Sciences (SIHAS), Sam Higginbottom University of Agriculture, Technology & Sciences (SHUATS), Allahabad, India

**Keywords:** Diazepam, Stress, Cardiac biomarkers, Ionic balance, Biochemical parameters, Cardiac dysfunction

## Abstract

Diazepam is a medicine of the family benzodiazepine, used to treat various CNS disorders. To date, no study is available for biochemical analysis of diazepam in cardiac dysfunction. This study aimed to determine the effect of diazepam in stress-induced cardiac dysfunctions in rats. Male Wistar Albino rats were divided into four groups with six animals in each group for 90 days of the experimental protocol. Group1 served as a Normal Control (NC), Groups 2, as a Disease Control (DC), Group 3 as a Diazepam Control (DIC), and Group 4 as a Disease + Diazepam Treatment (DDT). Disease Control and Disease + Diazepam Treatment animals exposed to regular stress by forced swimming exercise method for 3 months. Diazepam Control and Disease + Diazepam Treatment received 5 mg/kg/p.o the daily dose of diazepam. At the end of the protocol, animals were sacrificed, heart preserved, blood collected, and utilized for biochemical estimations. Heart weight was increased in DC as compared to NC. Serum levels of cardiac biomarkers, creatine phosphokinase (CPK), creatine kinase-MB (CPK-MB), lactate dehydrogenase (LDH), High sensitivity C-reactive protein (hs-CRP) and troponin I (TnI) were significantly increased in DC as compared to NC. Heart tissue examined for histological changes. The altered serum levels of CPK, CPK-MB, LDH, hs-CRP, and TnI were significantly restored by the treatment of diazepam. Serum levels of Sodium, Potassium, Calcium, and Magnesium was increased in DC animals as compared to NC. The altered ionic level was also restored by the treatment of diazepam. Level of various cardiac markers and ions in the plasma were also slightly elevated in DIC. Histopathological studies are also in agreement with serological examinations and bonafide cardioprotective influences of diazepam in cardiac dysfunction. Conclusively research findings endorse the cardioprotective effect of diazepam in stress-induced cardiac dysfunction in rats.

## Highlights

• Evaluation of diazepam in stress-induced cardiac dysfunction in rats.
• Alteration of cardiac biomarkers and ionic concentrations by stress.
• Restoration of altered cardiac biomarkers and ionic concentrations by diazepam.
• Restoration of architectures of cardiomyocytes by diazepam.

## Graphical abstract

**Figure:**
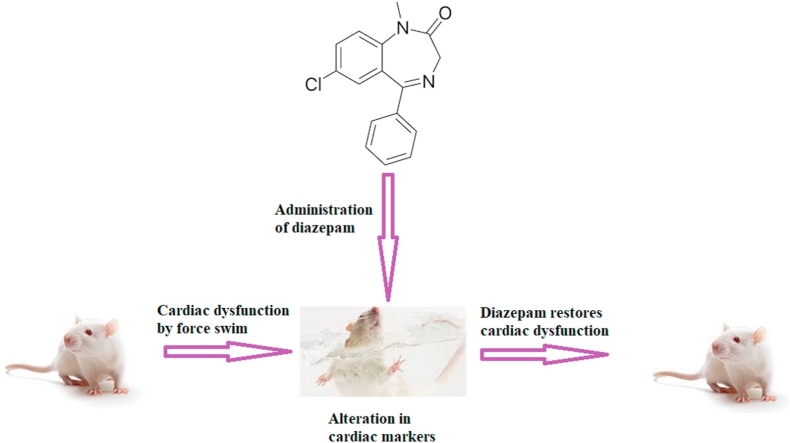

## 1 Introduction

Cardiovascular disorders is a main health issue of concern, as it covers the major economic burden of worldwide. It accounts for greater than half of overall mortalities in the developed nations [1]. The cardiovascular disorders incidence is mainly linked with some risk factors like lack of physical activity, smoking, overweight, hypertension, stress, hypercholesterolemia, hyperinsulinemia, to name a few [2,3]. It is one of the major causes of disability and mortality in the world. As per the study of Global Burden of Disease, stroke, and ischemic cardiac disease accounted for approximately 25 % casualty worldwide in 2013 [4].

Most of the people are, encountered with stressful life events or trauma many times during their life period, such as a diagnosis of severe health issues, life-threatening illness, death of family relatives and loved ones, violence, natural disasters, to name a few [5]. According to previous studies, such stress is one of the prominent factor may lead to induction of various diseases such as infection, injury, cardiovascular morbidity, psychiatric disorders, and several autoimmune diseases and death [6,7].

Stress linked disorders are a group of mental disorders induced by stressful life evens. Depending upon the intensity and type of stress, duration of stress, and various reflected symptoms, such disorders are mainly categorized as acute reactions of stress, adjustment disorder, and post-traumatic stress disorder [8,9]. Various life-threatening traumatic incidents are responsible for acute stress reaction, and post-traumatic stress disorder the former two disorders. However, adjustment disorders are mainly triggered by the significant and identifiable change in life which is also known as psychological or physical distress [10]. Post-traumatic stress disorder is the most extensively studied and severe type of stress disorder, which is identified by mood swings and fluctuations, avoidance, hyperarousal, and re-experiencing followed by the traumatic action [11,12]. The person exposed to Post-traumatic stress disorder has the risk of incidence of cardiovascular diseases. However, limited information is available on the role of Post-traumatic stress disorder in particular kinds of cardiovascular disease [13]. Further, the role of adjustment disorder, acute stress reaction disorder, and other stress linked disorders in cardiovascular disease development is almost unexplored [14]. In conclusion, cardiovascular disorders linked to various factors like lifestyle, genetic predisposition, as well as stress [15].

Diazepam, a benzodiazepine act by modifying GABA receptors [16], commonly used for several neurological disorders like seizures, anxiety, benzodiazepine withdrawal syndrome, alcohol withdrawal syndrome, sleeping disorder, restless legs syndrome and spasm of muscle [17,18]. It is also used for purposely loss of memory during various medical surgeries [19].

To date, no study is available on the protective effect of diazepam in stress-induced cardiac dysfunction. The role of diazepam in heart cells, is yet not clear, how it shows the effect on cardiac marker protein and ionic concentration in cardiac dysfunction. Hence, to evaluate the positive or negative effect of diazepam in stress exposed rats for cardiac dysfunction with the help of a cardiac marker and ionic balance, the present protocol was performed for investigation of effects of diazepam in stress-induced cardiac dysfunction in rats.

## 2 Materials and methods

### 2.1 Drugs and chemicals

Diazepam was procured from Sigma Aldrich Chemical, USA. Chemicals used in this study were procured from Himgiri Traders, Dehradun, Uttarakhand, India. Chemicals utilized in the experiment were from a commercial source and of analytical grade quality.

### 2.2 Animals

24 albino Wistar male rats approximate weight 140−160 g were procured from the Animal House facility of Department of Biochemistry, Science Faculty, King Abdulaziz University, Kingdom of Saudi Arabia. Animals were kept under appropriate climatic conditions, 24−27 °C with 12:12 cycle of light and dark, and fed with a good quality pellet diet. The experiment was approved and permitted for conduction by the Institutional Committee of Animal Ethics, Faculty of Science, King Abdulaziz University. Experimental procedures were performed with strict adherence to ethical guidelines and principles given by OECD guidelines (OECD 452, 2008; OECD 471, 2008B; ICH S2A 2008; ICH S2B, 1997).

### 2.3 Induction of cardiac dysfunction

For induction of cardiac dysfunction, Disease Control animals and Disease + Diazepam Treatment animals exposed to daily regular stress for half an hour by forced swimming exercise method for 3 months [20,21].

### 2.4 Experimental design

Male Wistar Albino rats were divided into 4 groups with 6 animals in each group for 90 days of the experimental protocol. Group I, was Normal Control (NC), Group 2 was Disease Control (DC), Group 3, Diazepam Control (DIC), and Group 4, Disease + Diazepam Treatment (DDT). For induction of cardiac dysfunction, DC and DDT Treatment animals exposed to daily regular stress for half an hour after feeding by forced swimming exercise method for 3 months. DIC and DDT received 5 mg/kg/p.o the daily dose of diazepam. At the end of the protocol, blood was collected from the tail vein. Before collection, the site cleansed with alcohol (70 %), kept under control, and then blood is withdrawn by using a needle of 21–22 gauge from the lateral vein of the tail. Quick after collection, the flow of blood was stopped with the application of pressure with sterile gauze for stopping blood flow [22]. Collected blood was centrifuged serum separated and processed for further biochemical study. After blood collection, animals were sacrificed and heart preserved for measurement of cardiac dysfunction.

### 2.5 Biochemical estimation

The serum levels of sodium (Na^+^), Potassium (K^+^), calcium (Ca^+^), magnesium (Mg^+^), creatine phosphokinase (CPK), creatine kinase-MB (CPK-MB), lactate dehydrogenase (LDH), High sensitivity C-reactive protein (hs-CRP) and troponin I (TnI) analyzed by utilizing a standard autoanalyzer kit (DimensionR RXL MAXTM, Siemens, Malvern, USA).

### 2.6 Histopathology

Rat hearts were isolated for histopathological analysis and fixed with 10 % buffered formalin, dehydrated by treating over an ordered sequence of alcohol with paraffin penetration. 5 μm sections of the tissue prepared by a rotatory microtome (semi-automated) and dried overnight at 37 °C. Hematoxylin and eosin staining done and observed at 40x magnification.

### 2.7 Statistical analysis

Data expressed expressed as Mean ± Standard error of mean. The significance value among different groups was calculated by one way analysis of variance and then student’s *t*-test was also used with Graph Pad Prism-5 software. The differences of p < 0.05 were considered as statistically significant.

## 3 Results

### 3.1 Heart weight

In Group 2 (DC) animals, heart weight significantly increased by forced swimming 840.1 ± 7.12 mg in comparison to Group 1 (NC) animals 480.2 ± 5.34 mg with the level of significance was p < 0.001 as compared to NC. Heart weight in Group 3 (DIC) animals was 505.5 ± 5.36 mg which as compared the to normal heart weight of NC animals 480.2 ± 5.34 mg with the level of significance was p < 0.01 as compared to DC animals. Weight of heart in Group 4 (DDT) animals was restored to the normal level by diazepam 515 ± 5.35 mg as compared to DC with the level of significance was p < 0.01 as compared to DC animals (Table 1).

**Table 1:** Effect of Diazepam on cardiac biomarkers in stress induced cardiac dysfunction in rats.

S.No	Name of groups	Heart Weight (mg)	CPK (IU/l)	CPK-MB (IU/l)	LDH (IU/l)	CRP (mg/dl)	Troponin I (ng/mL)
	Normal Control	480.2 ± 5.34	173.6 ± 1.23	90.23 ± 0.98	199.98 ± 2.03	09.74 ± 0.14	130.34 ± 1.36
	Disease Control	840.1 ± 7.12^###^	231.2 ± 2.31^##^	178.54 ± 1.61^###^	263.56 ± 2.56^##^	31.24 ± 0.26^###^	141.45 ± 1.44^##^
	Diazepam Control	505.5 ± 5.36**	175.3 ± 1.02**	92.98 ± 0.73**	205.45 ± 1.79***	10.01 ± 0.21***	136.33 ± 1.46**
	Disease + Diazepam Treatment	515 ± 5.35**	178.8 ± 2.03***	101.33 ± 1.213**	206.58 ± 1.99***	13.56 ± 0.79**	130.87 ± 1.08***

Values are expressed as mean ± SEM (N = 6). (^#^) Groups as compared to normal control; (*) Groups as compared to Disease Control; *P < 0.05; **P < 0.01; ***P < 0.001.

### 3.2 Creatine phosphokinase (CPK)

In Group 2 (DC) animals, the level of CPK significantly increased to (231.2 ± 2.31) IU/l in comparison to Group 1 (NC) animals (173.6 ± 1.23) IU/l. CPK level of Group 3 (DIC) animals was (175.3 ± 1.02) IU/l as compared to the normal level of NC animals (173.6 ± 1.23) IU/l. CPK level of Group 4 (DDT) animals was restored to the normal level with diazepam (178.8 ± 2.03) IU/l as compared to DC. The level of significance was p < 0.01 for DC and DIC animals while p < 0.001 in DDT animals (Table 1).

### 3.3 Creatine kinase-MB (CPK-MB)

In Group 2 (DC) animals, the level of CPK-MB significantly increased to (178.54 ± 1.61) IU/l in comparison to Group 1 (NC) animals (90.23 ± 0.98) IU/l. CPK-MB level of Group 3 (DIC) animals was (92.98 ± 0.73) IU/l which as compared to the normal level of NC animals. CPK-MB level of Group 4 (DDT) animals was restored to the normal level with diazepam (101.33 ± 1.213) IU/l as compared to DC. The level of significance was p < 0.001 for DC animals while p < 0.01 in DIT and DDT animals (Table 1).

### 3.4 Lactate dehydrogenase (LDH)

In DC animals, LDH level was significantly (p < 0.01) increased to (263.56 ± 2.56) IU/l in comparison to NC animals (199.98 ± 2.03) IU/l. LDH level of DIC animals was significant (p < 0.001) with normal value (205.45 ± 1.79) IU/l as compared to NC animals. LDH level of DDT animals was significantly (p < 0.001) restored to almost normal level (206.58 ± 1.99) IU/l as compared to DC (Table 1).

### 3.5 High sensitivity C-reactive protein (hs-CRP)

In DC animals, the level of hs-CRP was significantly (p < 0.001) increased to (31.24 ± 0.26) mg/dl in comparison to NC animals (09.74 ± 0.14) mg/dl. hs-CRP level of DIC animals was significant (p < 0.001) with normal value (10.01 ± 0.21) mg/dl as compared to NC animals. hs-CRP level of DDT animals was significantly (p < 0.01) restored near to normal level (13.56 ± 0.79) mg/dl as compared to DC (Table 1).

### 3.6 Troponin I (TnI)

In DC animals, the level of TnI was significantly (p < 0.01) increased to (141.45 ± 1.44) ng/mL in comparison to NC animals (130.34 ± 1.36) ng/mL. The TnI level of DIC animals was significant (p < 0.01) with normal value (136.33 ± 1.46) ng/mL as compared to NC animals. The TnI level of DDT animals was significantly (p < 0.001) restored near to normal level (130.87 ± 1.08) ng/mL as compared to DC (Table 1).

### 3.7 Sodium ions

In DC animals, sodium level was significantly (p < 0.001) increased to (203.36 ± 1.53) m.eq/l in comparison to NC animals (137.67 ± 1.89) m.eq/l. The sodium level of DIC animals was significant (p < 0.001) with normal value (163.34 ± 1.18) m.eq/l as compared to NC animals. The sodium level of DDT animals was significantly (p < 0.01) restored near to normal level (138.99 ± 1.39) m.eq/l as compared to DC (Table 2).

**Table 2:** Effect of Diazepam on ionic concentration in stress induced cardiac dysfunction in rats.

S.No.	Name of groups	Sodium (m.eq/l)	Potassium (m.eq/l)	Calcium (mg/dl)	Magnesium (mg/dl)
	Normal Control	137.67 ± 1.89	4.34 ± 0.18	9.94 ± 0.64	26.50 ± 0.16
	Disease Control	203.36 ± 1.53^###^	7.30 ± 0.23^##^	12.93 ± 0.85^##^	31.56 ± 0.47^##^
	Diazepam Control	163.34 ± 1.18***	5.76 ± 0.64**	11.41 ± 0.47**	26.31 ± 0.32***
	Disease + Diazepam Treatment	138.99 ± 1.39**	4.80 ± 0.28***	10.14 ± 0.16***	25.89 ± 0.26**

Values are expressed as mean ± SEM (N = 6). (^#^) Groups as compared to normal control; (*) Groups as compared to Disease Control; *P < 0.05; **P < 0.01; ***P < 0.001.

### 3.8 Potassium ions

In DC animals, the level of Potassium was significantly (p < 0.01) elevated to (7.30 ± 0.23) m.eq/l in comparison to NC animals (4.34 ± 0.18) m.eq/l. The potassium level of DIC animals was significant (p < 0.01) with normal value (5.76 ± 0.64) m.eq/l as compared to NC animals. The potassium level of DDT animals was significantly (p < 0.001) restored near to normal level (4.80 ± 0.28) m.eq/l as compared to DC (Table 2).

### 3.9 Calcium ions

In DC animals, the level of calcium was significantly (p < 0.01) increased to (12.93 ± 0.85) mg/dl in comparison to NC animals (9.94 ± 0.64) mg/dl. The calcium level of DIC animals was significant (p < 0.01) with normal value (11.41 ± 0.47) mg/dl as compared to NC animals. The calcium level of DDT animals was significantly (p < 0.001) restored near to normal level (10.14 ± 0.16) mg/dl as compared to DC (Table 2).

### 3.10 Magnesium ions

In DC animals, magnesium level was significantly (p < 0.01) raised to (31.56 ± 0.47) mg/dl in comparison to NC animals (26.50 ± 0.16) mg/dl. The magnesium level of DIC animals was significant (p < 0.001) with normal value (26.31 ± 0.32) mg/dl as compared to NC animals. The magnesium level of DDT animals was significantly (p < 0.01) restored near to normal level (25.89 ± 0.26) mg/dl as compared to DC (Table 2).

### 3.11 Histology

**Fig. 1:**
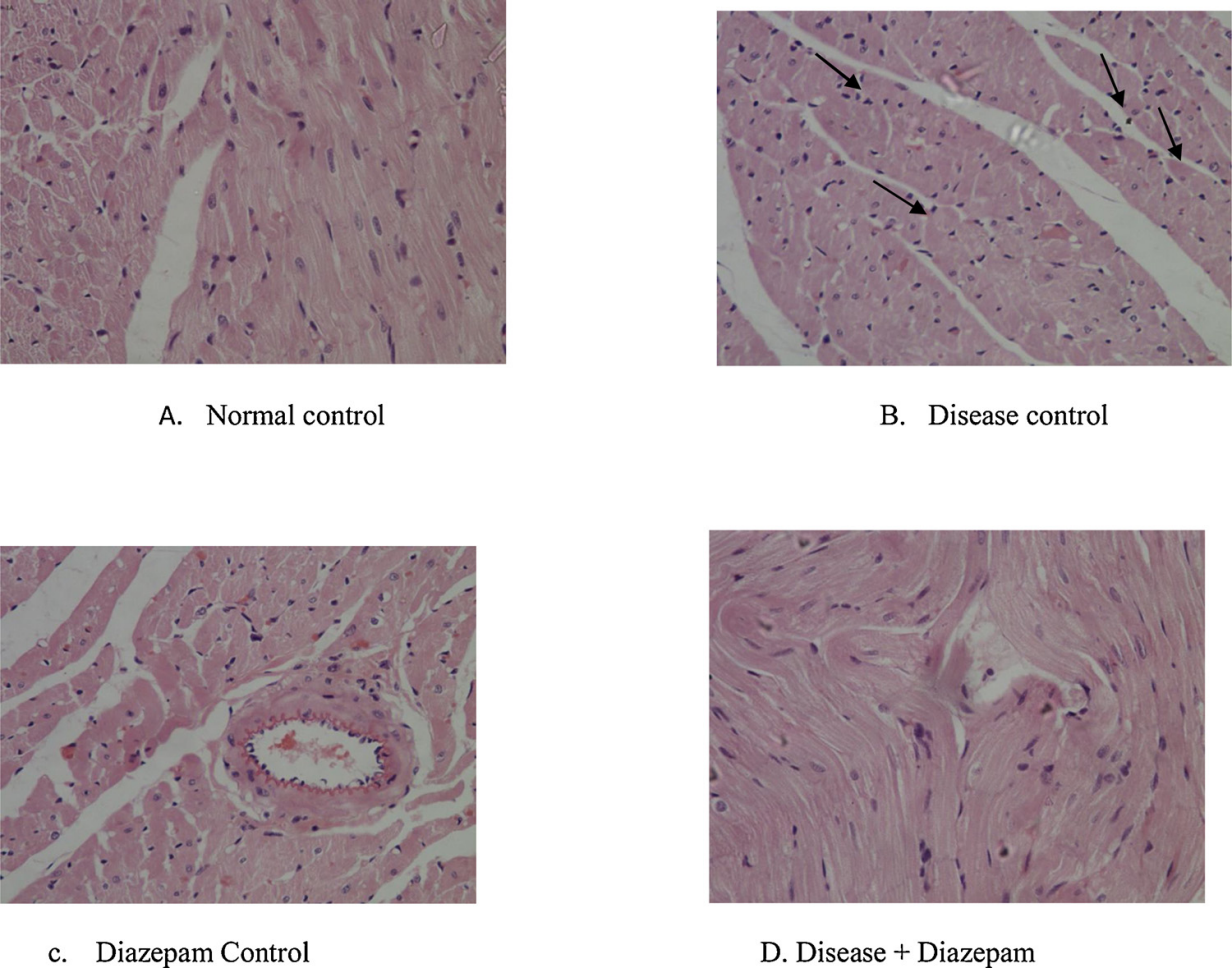
Showing histology of hearts. A.Normal control animals,B.Disease control animals,C.Diazepam control animals and D.disease + diazepam treated animals.

Fig. 1A illustrates the normal architecture of cardiocytes with normal tissue orientation, reduced deposits fats with no extra intercellular space. Disease control rats exhibited more interstitial gaps and abnormal intercalated discs (Fig. 1C), DDT treated group demonstrated less intercellular spaces normal architecture of cardiocytes with stratified cells, less irregular distribution of eosinophils and slight fat deposits (Fig. 1D).

## 4 Discussion

This study was designed for the evaluation of diazepam in cardiac dysfunction induced by force swimming or exercise model from your previous experiment. Forceful physical exercise leads to mental stress and the production of excess reactive oxygen species which is a leading cause of oxidative stress, which ultimately reflected in the form of dysfunction of the immune system, macromolecular oxidative damage, fatigue, and muscular damage [23]. Adaptation of heart to exercise training leads to functional, morphological, as well as electrical changes. The forceful exercise creates significant stress on the heart also. Lower ventricular hypertrophy is induced in such a case evident by increased lower ventricular mass, dimension, and wall thickness [24]. The efficacy of pumping of the heart depends upon the heart cavities size and morphological [25]. These changes can be confirmed by the estimation of different biochemical parameters [26].

In regular exercise, heat adapts itself according to the frequency, type, and extent of exercise and remodel it [27]. The duration of exercise is related to the changes in the morphology left ventricle which includes an increase in the thickness of the wall of the ventricle [28,29]. Further, due to force swimming, the physiology of the body is changed. Exercise leads to apnea to cover short distances underwater, which in turn could induce hypercapnia and hypoxia [30,31]. Forceful exercise leads to oxidative damage of lipoproteins of serum which may generate several disorders [32]. During such tremendous exercise, the various biochemical parameters of serum are altered and cause disease [33]. Further, oxidative stress leads to damage to DNA, which is directly linked with coronary heart artery disease. Damage of DNA is present in all the atherosclerotic plaque cells, which establishes the mechanism of the link between stress, DNA damage, and cardiovascular disease. Inside the mechanism of cardiac dysfunction oxidative stress plays a major role as a stimulant for transduction of signal in heart cells like MAP kinase and inflammation cytokines. Knowledge of the pathophysiological mechanisms in cardiac dysfunction and hypertrophy is important for new treatment plan development [34].

Stress plays a crucial role in cardiac dysfunction. With the increase of stress, the metabolic demands of heart cells increases, and at the same time stress exposure influences the performance of hart [35]. In athletes, chronic stress induces adaptation and heart enlargement [36]. But chronic stress many times induces stress-mediated myopathy of heart cells known as broken heart syndrome, which is identified by enlargement of the heart [37]. Due to this enlargement heart lose their ability of contraction, which ultimately leads to cardiac myopathy and injury of heart muscles [38]. Stress-induced changes may vary depending upon the mode of stress, gender, age, nutrition, race, genetic factors, physical activity, and psychological factors [39]. Considering the effect of chronic stress on heart, in this experiment forced swimming stress model chosen for the induction of cardiac dysfunction. Evidences from previous studies suggest that diazepam shows protective effects on heart. Diazepam increases myocardial oxygen supply and shows an oxygen conserving action in heart. This suggests diazepam is beneficial to the patient with coronary heart disease [40]. It is helpful in maintaining balance between oxygen flow and blood to heart [41]. Further, diazepam is helpful in maintaining blood pressure and heart rate in coronary artery disease patients [42]. To date only a few works available on the effect of diazepam on heart function. Most of the work available on cardiac dysfunction is on associated with chemical or inflammation induced cardio toxicity [[43], [44], [45]]. No work is available on the effect of diazepam on the cardiac marker and stress-induced cardiac dysfunction. The current study gives a complete idea about the effect of diazepam on biochemical parameters and cardiac marker which is associated with cardiac dysfunction.

In this study, diazepam restored DDT group animal heart size significantly (p < 0.01) to 515 ± 5.35 mg as compare to 840.1 ± 7.12 mg. Heart size is the main physical parameter for the assessment of cardiac dysfunction [46]. The restoration of heart size by diazepam indicates, the cardioprotective potential in dysfunction. CPK is also popular as creatine kinase. CPK is an enzyme that phosphorylates the creatine [47]. It is present in cardiomyocytes. In myocytes, it has a crucial role in chemical energy transport in fulfilling the demands of the cardiac cells [48]. It is one of the important cardiac marker enzymes used for the determination of heart disease and chest pain [49]. The higher level of CPK in the blood indicates the damage of CPK rich cells and tissue-like in myocardial infarction, rhabdomyolysis, myocarditis, and myositis [50]. CPK in the blood may be increased in various diseases including cardiac dysfunction, hypothyroidism, statins use, neuroleptic malignant syndrome, and malignant hyperthermia [51]. In our study, the level of CPK was restored significantly (p < 0.001) to (178.8 ± 2.03) IU/L in comparison to DC (231.2 ± 2.31) IU/l animals. This indicates that diazepam successfully restored the altered CPK level and shows the cardioprotective effect in cardiac remodeled rats.

CPK-MB is one of the most specific and indicator diagnoses of various heart disorders like myocardial infarction. Level of CK-MB isoenzyme increases in different king of heart disease [52]. In this study level of CPK-MB was significantly (p < 0.001) increased (178.54 ± 1.61) IU/l, which indicates cardiac injury and dysfunction in rats by stress. This abnormal level was restored significantly (p < 0.001) to the normal value (101.33 ± 1.213) IU/l by diazepam, which reflects diazepam prevents cardiac injury and dysfunction. LDH is an enzyme that is widely distributed in the heart and other tissues. This enzyme metabolizes pyruvate to lactate in a short oxygen supply. The increased level of serum LDH is an indicator of necrosis, tissue injury, hemolysis, hypoxia, and heart disease. In our study, an elevated level of LDH (263.56 ± 2.56) IU/l found in DC animals, which indicates cardiac injury and dysfunction, induced in DC animals. Further diazepam decrease elevated level to normal (206.58 ± 1.99) IU/l, clearly indicate diazepam shows a protective effect against heart disease. CRP is a protein that increases in the serum with infection, inflammation surgery, heart attack, other heart disease, and trauma [53]. In this study, in DC animals the level of CRP increased (31.24 ± 0.26) mg/dl, which confirms cardiac disorder in induced. Elevated level as successfully lowered to the normal value (13.56 ± 0.79) mg/dl by diazepam, confirms the ameliorative efficacy of diazepam. TnI is a skeletal muscle and cardiac protein used in the diagnosis of heart injury and heart attack. Level of Tnl, increase in heart injury. In the current study [54], the Tnl level was increased (141.45 ± 1.44) ng/mL in DC animals and this elevated level was significantly (p < 0.001) restored to normal level (130.87 ± 1.08) ng/mL. The restoration of abnormal levels of Tnl indicates the cardioprotective potential of diazepam.

The level of sodium, potassium, calcium, and magnesium is directly associated with the normal functioning of the heart [55,56]. An imbalance of these ions in the body leads to dysfunction of the ionic channel of heart which is one of the most important underlying mechanisms of the cardiac dysfunction, arrhythmias, and reduced heart muscle contractility [57]. Cardiac dysfunction and associated abnormality of the heart are linked with changes in signaling, structural, metabolic, and regulatory proteins [58]. Ionic channels, pumps, and transporters are only a part of proteins which altered in cardiac disorder. However, as the main regulator of membrane contraction and excitation, they are the main key targets in restoring heart disease [59]. Abnormal levels of sodium, potassium, calcium, and magnesium result in uniformly and consistently decreased heart muscle contraction capacity, increased arrhythmias incidence, and other different cardiac disorders [60]. Hence, the restoration of ionic imsbalance is very important in reversing cardiac disorder. In our study, the level of sodium, potassium, calcium, and magnesium was increased significantly (p < 0.01 and p < 0.001) in DC animals. This indicates that regular stress completely altered the ionic level of the body and induced heart disorder and cardiac dysfunction. This altered ionic level was significantly (p < 0.01 and p < 0.001) restored by diazepam. It means diazepam prevent cardiac dysfunction and injury and has a strong cardioprotective effect.

## 5 Conclusion

From the outcomes of results, it can be suggested that diazepam shows a cardioprotective effect in stress-induced cardiac remodeled rats. It shows cardioprotection by the restoration of heart size, cardiac marker enzymes, and ionic balance in stress-induced cardiac remodeled rats. The further clinical study required to explore this finding in cardiac dysfunction patients.

### Authors’ contributions

FAA designed and performed the work, VK revised manuscript whereas FA performed data analysis and prepared manuscript.

## CRediT authorship contribution statement

**Fahad A. Al-Abbasi:** Conceptualization, Methodology, Software. **Vikas Kumar:** Data curation, Writing - original draft. **Firoz Anwar:** Visualization, Investigation, Supervision, Software, Validation, Writing - review & editing.

## Declaration of Competing Interest

The authors of this manuscript have no conflict of interest and responsible for the complete writing content of this research paper.
